# The Crystal Structure of PPIL1 Bound to Cyclosporine A Suggests a Binding Mode for a Linear Epitope of the SKIP Protein

**DOI:** 10.1371/journal.pone.0010013

**Published:** 2010-04-02

**Authors:** Christian M. Stegmann, Reinhard Lührmann, Markus C. Wahl

**Affiliations:** 1 Max-Planck-Institut für biophysikalische Chemie, Zelluläre Biochemie/Makromolekulare Röntgenkristallographie, Göttingen, Germany; 2 Max-Planck-Institut für biophysikalische Chemie, AG Strukturbiochemie, Institut für Chemie und Biochemie, Freie Universität Berlin, Berlin, Germany; Griffith University, Australia

## Abstract

**Background:**

The removal of introns from pre-mRNA is carried out by a large macromolecular machine called the spliceosome. The peptidyl-prolyl *cis/trans* isomerase PPIL1 is a component of the human spliceosome and binds to the spliceosomal SKIP protein *via* a binding site distinct from its active site.

**Principal Findings:**

Here, we have studied the PPIL1 protein and its interaction with SKIP biochemically and by X-ray crystallography. A minimal linear binding epitope derived from the SKIP protein could be determined using a peptide array. A 36-residue region of SKIP centred on an eight-residue epitope suffices to bind PPIL1 in pull-down experiments. The crystal structure of PPIL1 in complex with the inhibitor cyclosporine A (CsA) was obtained at a resolution of 1.15 Å and exhibited two bound Cd^2+^ ions that enabled SAD phasing. PPIL1 residues that have previously been implicated in binding of SKIP are involved in the coordination of Cd^2+^ ions in the present crystal structure. Employing the present crystal structure, the determined minimal binding epitope and previously published NMR data [1], a molecular docking study was performed. In the docked model of the PPIL1·SKIP interaction, a proline residue of SKIP is buried in a hydrophobic pocket of PPIL1. This hydrophobic contact is encircled by several hydrogen bonds between the SKIP peptide and PPIL1.

**Conclusion:**

We characterized a short, linear epitope of SKIP that is sufficient to bind the PPIL1 protein. Our data indicate that this SKIP peptide could function in recruiting PPIL1 into the core of the spliceosome. We present a molecular model for the binding mode of SKIP to PPIL1 which emphasizes the versatility of cyclophilin-type PPIases to engage in additional interactions with other proteins apart from active site contacts despite their limited surface area.

## Introduction

Peptidyl-prolyl *cis/trans* isomerases (PPIases, E.C. 5.1.2.8) catalyze the isomerization of peptide bonds that precede a proline residue and can thereby facilitate folding of their substrate proteins [2]. PPIases are implicated in a number of cellular processes including transcriptional control and pre-mRNA splicing [3]. These enzymes have been documented in all three domains of life and have recently also been found in a viral genome [4]. The PPIases belong to three classes: cyclophilins (cyclosporine A binding proteins, Cyps), FK506-binding proteins and parvulins. Proteomics analyses of spliceosomes have revealed that eight cyclophilins are present in the human spliceosome (reviewed by [5] and [6]). The abundance of these enzymes in pre-mRNA splicing in humans is striking and raises the question as to their contributions to the splicing cycle. The splicing cycle comprises the ordered assembly of the spliceosome on its pre-mRNA substrate, catalysis of the splicing reaction as well as the disassembly and recycling of its components. Specifically, the assembly is characterized by the stepwise recruitment of small nuclear ribonucleoprotein particles (snRNPs) as well as non-snRNP proteins. It is initiated by U1 snRNP binding to the 5′-splice site (complex E) followed by U2 snRNP recognizing a conserved branch point sequence in the intron (complex A). Subsequently, a pre-formed U4/U6-U5 tri-snRNP is integrated, giving rise to the pre-catalytic complex B spliceosome, which is still inactive. This particle undergoes compositional and conformational rearrangements to become catalytically competent for the first step, resulting in complex B*. Complex B* carries out the first transesterification reaction. Afterwards, remodeling generates a spliceosome that catalyzes the second step (complex C). Finally, the post-catalytic complex is disassembled in an ordered fashion.

Peptidyl-prolyl isomerase like 1 (PPIL1) is a cyclophilin and belongs to the so-called Prp19-related proteins [5]. It is recruited into the spliceosome at the stage of complex B formation. It was recently defined as a core component of complex C, since it remains in the complex even after treatment with 1 M NaCl [7]. PPIL1 is a single domain cyclophilin and has been found to interact with the Ski-interacting protein (SKIP, also referred to as hPrp45, SNW domain-containing protein 1, nuclear receptor coactivator NCoA-62). SKIP itself is a Prp19-related protein [5] that is stably integrated into the C complex [7] and whose ortholog in *Saccharomyces cerevisiae*, Prp45, is essential for pre-mRNA splicing [8]. In addition to its presence in splicing complexes, SKIP has also been found to act as a co-activator of Notch-, VDR- and SMAD-regulated transcription (see [9] for review) and was therefore thought to be a factor that links transcription and splicing. However, more recent data suggest that the roles of SKIP in transcription and splicing are independent [10].

PPIL1 is up-regulated in human colon cancer cells and an siRNA-mediated knockdown of PPIL1 resulted in a reduced number of viable cells [11]. Analogous to the binding mode of the spliceosomal hPrp4 protein to cyclophilin H [12], the PPIL1·SKIP interaction does not occupy the PPIase active site [1], [13], which suggests that SKIP serves as an adaptor for the cyclophilin. This finding has led to the speculation that SKIP and PPIL1 could participate in the activation of the spliceosome by SKIP delivering the cyclophilin to a site where remodeling is required [9]. However, in contrast to SKIP, human PPIL1 does not have an ortholog in *S. cerevisiae*.

Here, we investigated the PPIL1 protein biochemically and structurally. We could delineate a minimal PPIL1-binding epitope of the SKIP protein sufficient to pull-down PPIL1 protein. An atomic resolution crystal structure of PPIL1 in complex with the inhibitor CsA, the identification of the minimal PPIL1-binding epitope of SKIP and previously published NMR data [1] enabled us to carry out a molecular docking study. The docked model of the PPIL1·SKIP interaction suggests a novel protein-binding surface of a cyclophilin.

## Results

### Identification of a PPIL1-binding SKIP epitope by a peptide array

Based on its amino acid sequence, several algorithms predict that the SKIP protein is intrinsically unstructured to a large extent (not shown). We therefore speculated that it may interact with PPIL1 *via* a linear binding epitope. Xu *et al.* [1] could narrow down SKIP residues that interact with PPIL1 to a region comprising amino acids 59 to 129 by a GST pull-down experiment. In order to map the PPIL1-interacting region of SKIP at single amino acid resolution, a peptide array analysis was performed. A total of 73 20-mer peptides covering SKIP residues 49 to 140 with a one amino acid offset were synthesized in duplicate as a spot array on cellulose membranes (kind gift of Dr. C. Freund, FMP, Berlin, Germany).

**Figure 1:**
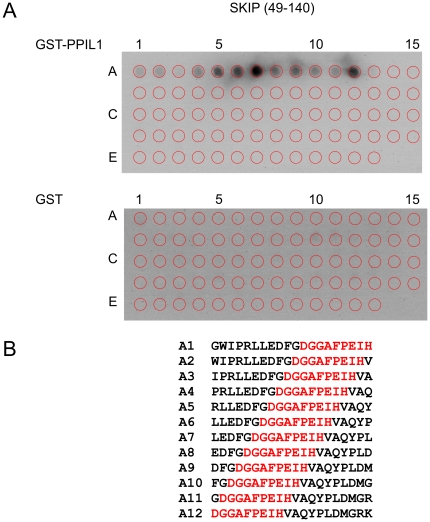
PPIL1 binds short epitopes of SKIP in peptide scanning experiments. (A) Peptide scan analysis of the PPIL1·SKIP interaction. Cellulose-bound peptide arrays were successively incubated with GST-PPIL1 fusion protein (upper panel) or GST alone (lower panel), rabbit α-GST antibody and horseradish peroxidase-coupled goat α-rabbit antibody. Images were obtained after luminol reaction. The locations of the spotted peptides are indicated by red circles. (B) Binding peptides exhibiting luminol signal above background. The consensus sequence common to all binding peptides is highlighted.

Recombinant GST-PPIL1 bound strongly to a stretch of consecutive peptides in the array (Figure 1A, upper panel), indicating a linear PPIL1-binding epitope on SKIP. A control experiment with GST alone did not show significant signal above the background (Figure 1A, lower panel). The minimal binding sequence, present in all interacting peptides, is ^61^GGAFPEIH^68^ (Figure 1B).

### GST pull-down experiments confirm the mapped SKIP epitope

**Figure 2:**
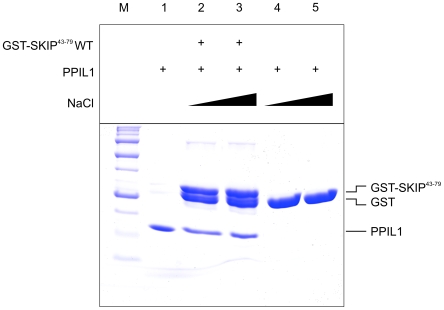
SKIP43–79 is sufficient to pull-down PPIL1. Pull-down of purified PPIL1 with glutathione-sepharose beads pre-coated with recombinant GST-SKIP^43–79^ (lanes 2 and 3) shows that PPIL1 binds to the mapped SKIP epitope, while GST alone (lanes 4 and 5) does not bind PPIL1. NaCl concentration in the washing buffer was varied between 150 mM (lanes 1, 2 and 4) and 1 M (lanes 3 and 5). Input (lane 1) and bound fractions (lanes 2 to 5) were analyzed by SDS-PAGE and stained with Coomassie. The band below the GST-SKIP fusion protein in lanes 2 and 3 is likely an abortive translation product corresponding to GST alone.

To confirm the binding epitope as mapped by the peptide array experiment, a GST-fusion construct containing SKIP residues 43–79 was employed for GST pull-down experiments using purified PPIL1 protein. This construct encompasses a longer SKIP-polypeptide than the minimal epitope derived from the peptide array in order to allow formation of short secondary structure elements neighboring the immediate interaction region, as previously seen in the CypH-binding loop of hPrp4 [12]. Figure 2 shows that the mapped epitope comprising SKIP residues 43 to 79 fused to GST is sufficient to pull-down purified PPIL1, while GST alone does not pull-down PPIL1, indicating a specific binding reaction. The interaction persists under stringent washing conditions (1 M NaCl and 0.1% (w/v) Triton X-100). However, the complex was unstable during size exclusion chromatography (not shown), suggesting kinetic instability of the complex.

### Crystal structure of PPIL1 bound to cyclosporine A

**Figure 3:**
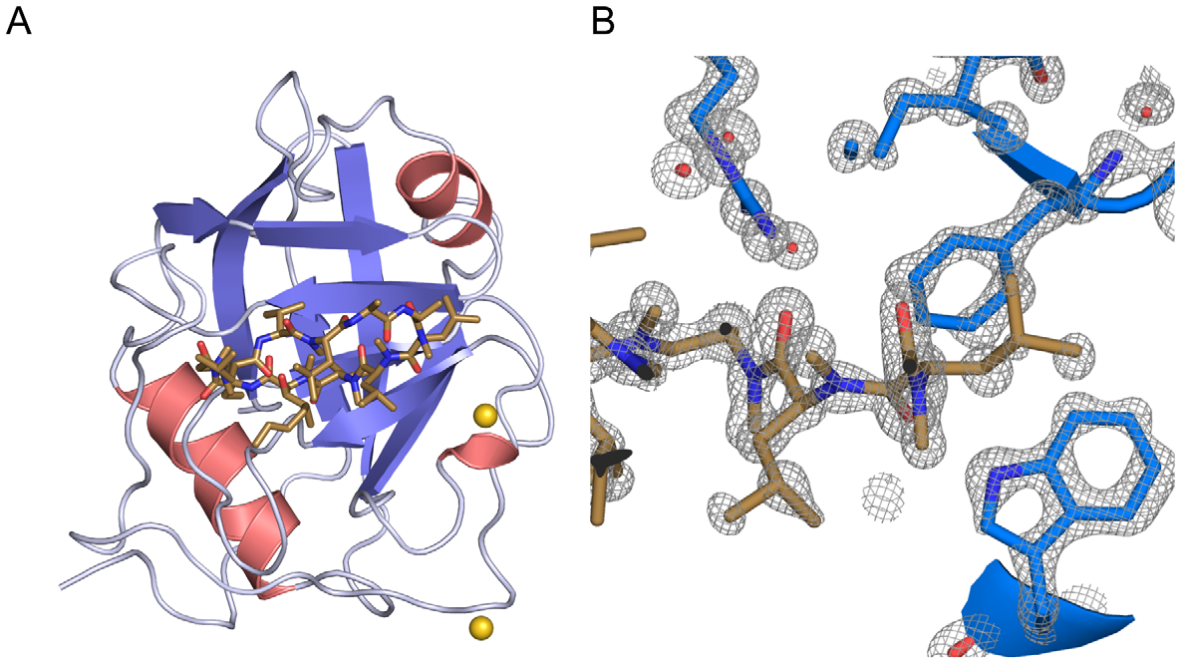
Crystal structure of the PPIL1·CsA complex. (A) Cartoon representation of PPIL1 with bound CsA (carbon atoms: brown) and two Cd^2+^ ions (golden spheres). (B) Experimental electron density obtained by SAD phasing contoured at the 2σ level (grey mesh). The final model is displayed as sticks.

PPIL1 protein was subjected to crystallization trials in the presence of synthetic peptides encompassing SKIP residues 55–74 and 44–74 (synthesized by Peptide Specialty Laboratories, Heidelberg, Germany). To sample a larger crystallization parameter space, the active site inhibitor CsA was included in a set of samples. Crystals were obtained in samples containing both SKIP 44–74 peptide and CsA and grew as clusters of rectangular plates. Singularized crystals diffracted to a resolution of 1.15 Å and exhibited two bound Cd^2+^ ions that enabled SAD phasing (Table 1). The experimental electron density was of excellent quality (Figure 3B) and permitted automated model building with Arp/wArp for 90% of the model. The final model comprises full-length PPIL1 protein (except the very N-terminal Met) bound to CsA without any geometry violations and with good R-factors (Table 1). However, neither the experimental electron density map nor the map obtained after refinement showed electron density for a bound SKIP peptide. The lack of electron density for the SKIP-peptide indicates that the peptide, although present in twofold molar excess in the crystallization setup, was excluded from the crystal during nucleation and growth. At a later stage, using refined crystallization conditions, morphologically similar crystals could be produced with PPIL1·CsA complex lacking the SKIP peptide, suggesting that the SKIP peptide was not necessary for crystallization.

**Table 1:** Crystallographic data and refinement.

Data Collection		
	Set1 (phasing)	Set2 (high resolution)
**Wavelength** (Å)	1.5	0.9
**Temperature** (K)	100	100
**Space Group**	P21212	P21212
**Unit Cell Parameters** (Å)	103.2 35.7 45.9	103.2 35.7 45.9
**Resolution** (Å)	50.0−1.79 (1.89−1.79)^a^	50.0−1.15 (1.25−1.15)
**Reflections**		
Unique	16,563	62,933
Completeness (%)	99.9 (99.5)	99.8 (99.4)
Redundancy	14.6 (14.5)	6.7 (6.1)
**Mean I/σ(I)**	19.2 (5.6)	8.9 (2.0)
**R_sym_(** ***I*** **)** b	11.9 (45.8)	8.6 (44.4)

Phasing		
**Resolution** (Å)		2.5
**Heavy Atom Sites**		2
**Correlation Coefficients** c		
SHELXD CC/CC_weak_		39.0/24.9
Pseudo-Free CC Left/Right Hand		82.0/55.1

Refinement		
**Resolution** (Å)		35.0−1.15
**Reflections**		
Number		61,160
Completeness (%)		99.9
Test Set (%)		5
**R_work_** d (%)		12.8
**R_free_** d (%)		14.9
**ESU** (Å)^e^		0.09
**Contents of A.U.** f		
Protein Molecules/Residues/Atoms		1/167/3138
Water Oxygens		294
Ligand Molecules/Atoms		1/191
**Mean B-Factors** (Å^2^)		
Wilson		8.13
Protein		12.3
Water		27.8
Ligand		14.3
**Ramachandran Plot** g		
Preferred		162
Allowed		6
Disallowed		-
**r.m.s.d.** h **from Target Geometry**		
Bond Lengths (Å)		0.009
Bond Angles (°)		2.20
Chirality (Å)		0.074
Dihedral Angles (°)		17.997
**PDB ID**		2X7K

a Data for the highest resolution shell in parentheses.

b R_sym_(*I*) = Σ_hkl_Σ_i_∣*I*_i_(hkl)−<*I*(hkl)>∣/Σ_hkl_Σ_i_∣*I*_i_(hkl)∣; for *n* independent reflections and *i* observations of a given reflection; <*I*(hkl)> – average intensity of the *i* observations.

c CC = [Σ*wE*_o_ *E*_c_Σ*w*−Σ*wE*_o_Σ*wE*_c_]/{[Σ*wE*_o_^2^Σ*_w_*−(Σ*wE*_o_)^2^] [Σ*wE*_c_^2^Σ*w*−(Σ*wE*_c_)^2^]}^½^; *w* – weight (see http://shelx.uni-ac.gwdg.de/SHELX/shelx_de.pdf for full definitions).

d R = Σ_hkl_∣∣*F*_obs_∣−∣*F*_calc_∣∣/Σ_hkl_∣*F*_obs_∣; R_work_−hkl ∉ T; R_free_−hkl ∈ T; R_all_ – all reflections; T – test set.

e ESU – estimated overall coordinate error based on maximum likelihood.

f A.U. – asymmetric unit.

g According to [26].

h Rmsd – root-mean-square deviation.

PPIL1 exhibits the canonical cyclophilin fold comprising an eight-stranded antiparallel β-sheet capped on both sides by α-helices (Figure 3A). The secondary structure of the present PPIL1 is identical to that of the archetypical cyclophilin, CypA [14], [15]. As known from other cyclophilins, the active site is located on strands β3 and β4. All CsA-contacting residues of PPIL1 are conserved and exhibit the same side chain rotamers as observed for CypA in complex with CsA, rendering the active site geometry essentially identical to CypA and other CypA-type cyclophilins.

### Cadmium ions mediate crystal contacts

**Figure 4:**
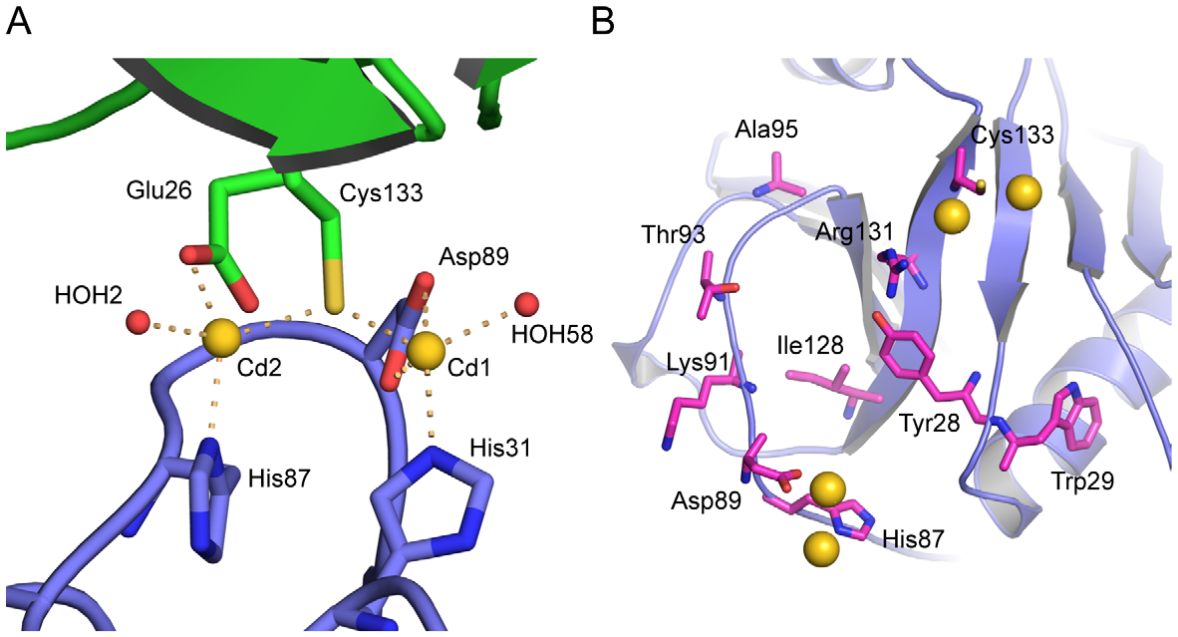
Cadmium ions are coordinated by two neighboring PPIL1 molecules and solvent molecules. (A) Cd1 (gold sphere) is coordinated by Asp89 in a bidentate fashion and by His31 of one PPIL1 molecule (carbon atoms in light blue) as well as by Cys133 of the (1/2-x, y-1/2, -z)-symmetry-related molecule (carbon atoms in green). The fourth coordination is site taken by a solvent molecule (red sphere). Cd2 shown here is (1/2-x, y-1/2, -z)-symmetry-related and coordinated by His87 of the present molecule and Glu26 and Cys133 of the (1/2-x, y-1/2, -z)-symmetry-related molecule as well as a solvent molecule. (B) Carbon atoms of PPIL1 residues that have been previously mapped to interact with a SKIP peptide by HSQC-NMR [1] are shown in magenta. These residues include the Cd^2+^-coordinating residues His87, Asp89 and Cys133.

The structure of the PPIL1·CsA complex contains two Cd^2+^ ions in an asymmetric unit. Both ions are coordinated by the PPIL1 protein and water molecules such that contacts between two neighboring PPIL1 molecules in the crystal lattice are established (Figure 4A). Cd1 is complexed in a distorted penta-coordinate manner by His31, Asp89, a solvent molecule and the symmetry-related Cys133. The carboxylate group of Asp89 acts as a symmetrical bidentate ligand for Cd1, which can alternatively be viewed as a single ligand centered at the position of its carbon [16]. This binding mode yields a slightly distorted tetrahedral coordination geometry with a mean angle of 109.0° (standard deviation 9.3°, Table S1). In constrast, Cd2 is coordinated by the carboxylate group of Glu26 in a monodentate fashion. The other ligands of Cd2 are Cys133, a solvent molecule and the symmetry-related His87, resulting in a distorted tetrahedral geometry (mean angle 108.6°, standard deviation 14.2°, Table S1). The bridging Cys133 coordinates both Cd^2+^ ions at almost equivalent distances (2.54 Å and 2.49 Å respectively), resulting in a Cd1–Cys133Sγ–Cd2 angle of 111.2°. These intricate interactions readily explain why PPIL1·CsA crystals could only be grown in the presence of CdCl_2_. Like zinc, cadmium possesses a filled d^10^ orbital shell and is therefore able to accommodate different coordination geometries of similar energy. The tendency of cadmium as a soft transition metal to bind to thiolate groups is likely the reason why cadmium could not be substituted by other divalent transition metals.

Using HSQC-NMR experiments, Xu *et al.* [1] were able to pinpoint a number of PPIL1 surface-exposed residues that undergo chemical shift perturbations upon titration with SKIP59–129. Strikingly, these residues (highlighted in Figure 4B) involve the residues His87, Asp89 and Cys133, which coordinate Cd^2+^ ions in the present crystal structure. In addition, all other presumed SKIP-binding residues [1] are in close vicinity of the Cd^2+^ binding site. This finding suggests that Cd^2+^-mediated crystal packing presumably excluded the SKIP peptide during crystallization.

### Molecular docking suggests a model for the PPIL1·SKIP interaction

Lacking crystallographic data of the SKIP peptide interacting with PPIL1, an *in silico* molecular docking study was performed using the High Ambiguity Driven protein-protein Docking algorithm (HADDOCK, [17]). The coordinates for the PPIL1 protein were taken from the current crystal structure without modifications, while coordinates for a ten-residue SKIP peptide were modeled *ab initio* (see Materials and Methods). Restraints for docking were based on experimental data of the PPIL1-binding epitope of SKIP as mapped by peptide array analysis and SKIP-interacting residues as mapped by HSQC-NMR by [1].

Solutions were clustered automatically and resulted in a single cluster of docking results, which stood out among the remaining clusters: The score was −40.5±8.6 for the best-scoring cluster, whereas next best clusters had scores of −27.4±10.1, −26.8±4.1 and −26.5±4.8 respectively. Likewise, all cluster evaluation parameters showed a significantly better performance of the best-scoring cluster versus the remaining clusters. For example, structures from the best-scoring cluster showed the smallest r.m.s.d. from the overall lowest-energy structure (0.5 Å±0.3 Å) and the largest buried surface areas (728.3 Å^2^±106.4 Å^2^), suggesting that solutions of the best-scoring cluster present sensible models for the interaction.

**Figure 5:**
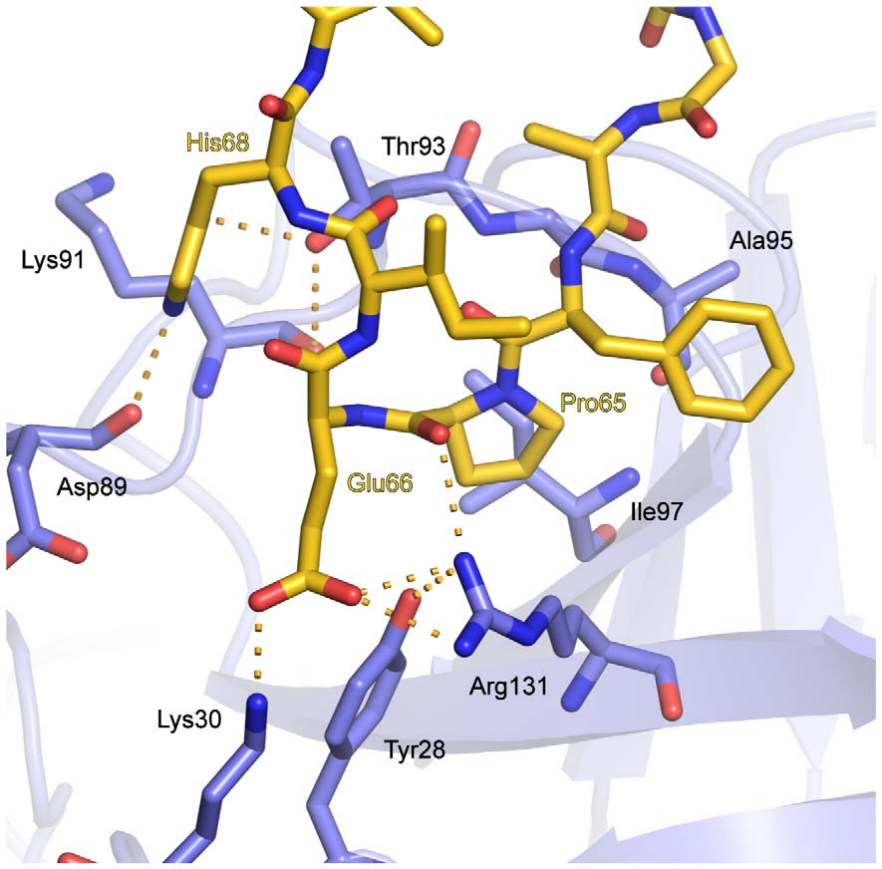
A docked model for the PPIL1·SKIP interaction. The SKIP peptide (carbon atoms gold) adopts a loop structure, in which the central Pro65 is buried in a hydrophobic groove formed by Ile97 and the aliphatic region of the side chain of Arg131 of PPIL1 (PPIL1 carbon atoms light blue). The Glu66 side chain of the SKIP peptide engages in hydrogen bonds to PPIL1 residues Tyr28, Lys30 and Arg131. Furthermore, His68 of SKIP is bound by Asp89 and Thr93 of PPIL1.

Figure 5 shows the lowest-energy structure of the best-scoring cluster of the HADDOCK docking run. In this model, the SKIP peptide adopts a loop structure sliding Pro65 into a hydrophobic groove formed by Ile97 and the aliphatic region of the side chain of Arg131 of PPIL1. The acidic Glu66 side chain of the SKIP peptide engages in hydrogen bonds/ion bridges to three PPIL1 residues, *i.e.* Tyr28, Lys30 and Arg131. Furthermore His68 of SKIP is bound by Asp89 and Thr93 of PPIL1. Overall, the SKIP peptide adopts a conformation that allows a snug fit into a pocket of PPIL1, which is essentially unchanged compared to the Cd^2+^-ion dependent crystal structure of PPIL1 (r.m.s.d. for docked PPIL1 to crystal structure is 0.45 Å).

## Discussion

### PPIL1 binds to a short, linear SKIP epitope

PPIL1 is a spliceosomal cyclophilin that possesses two striking features among the eight known human spliceosomal cyclophilins. First, PPIL1 is one of two cyclophilins that is tightly integrated into the human spliceosomal C complex (as is CypE) as evidenced by high-salt treatment and gradient centrifugation [7]. Second, PPIL1 can interact with the SKIP protein without involving its PPIase active site [1], [13]. A peptide scan revealed that a minimal binding epitope consisting of SKIP amino acids 61 to 68 is sufficient for PPIL1 binding. This epitope is also contained in a GST-SKIP^43–79^ construct that can efficiently pull-down PPIL1. The GST-SKIP^43–79^·PPIL1 interaction is stable in high-salt conditions, which argues for SKIP functioning as an adaptor protein that integrates PPIL1 into the high-salt-resistant core of the human spliceosome. SKIP is predicted to be intrinsically unstructured; therefore it could undergo a disorder-to-order transition upon integration into the spliceosome and thereby deliver PPIL1 to a specific site where PPIase activity is required. *In vitro*, the PPIL1·SKIP interaction is kinetically unstable and the complex disintegrates during size exclusion chromatography.

### Docking of a SKIP peptide to the PPIL1·CsA crystal structure suggests that binding occurs *via* a novel binding interface

The present crystal structure showed full-length PPIL1 protein in complex with CsA but did not reveal electron density for the SKIP peptide that was added in two-fold molar excess. Instead, PPIL1 residues that have been mapped by HSQC-NMR to interact with SKIP [1] are coordinating Cd^2+^ ions. Cd^2+^ ions are directly involved in crystal packing and it appears, thus, that Cd^2+^-mediated crystal packing has excluded the SKIP peptide from the crystals. The Cd^2+^ ions are coordinated at a distinct site of the protein that comprises residues in the EF-loop of the cyclophilin (Asp89, Lys91) as well as Tyr28 of the BC-loop and Arg131 of the βI sheet. It is noteworthy that these residues are not conserved among cyclophilins (e.g. Asp89 appears to be unique among spliceosomal cyclophilins; Figure S1). Furthermore, these residues are spatially distinct from the hPrp4-binding site of CypH, which is primarily located on its extended CD-loop that forms a β-hairpin [12].

The PPIL1·CsA crystal structure exhibits a generally more compact shape compared to a previously published NMR structure ensemble of PPIL1[1]. In particular, the N-terminus of the protein is not disordered as reported for the NMR structure. It folds over the α1 helix by Ile4 engaging in van der Waals contacts with Tyr78 and Leu86.

### Model for the PPIL1·SKIP interaction

The present molecular docking study provides a model for the PPIL1·SKIP interaction, in which the SKIP peptide binds into a pre-formed pocket of PPIL1 that has not been observed in other cyclophilins before. The buried surface area of the complex is 728 Å±106 Å, which is comparable to the area buried in the case of the CypH·hPrp4 complex (878 Å).

Perhaps surprisingly, the PPIL1·SKIP interaction deduced herein involves a proline residue of SKIP buried in a hydrophobic pocket of the PPIL1 protein. The active site of cyclophilins also exhibits affinity for Pro-containing peptides, raising the question of why the SKIP-peptide selectively binds to the alternative pocket. Our model suggests that Glu66 of SKIP, neighboring the central Pro65, could act as a discriminator. The Glu66 carboxyl group of SKIP engages in polar contacts to the PPIL1 protein, showing that it is a critical anchoring point of the SKIP peptide.

During the finalization of this work, a study describing an NMR-ensemble of the PPIL1·SKIP interaction was published [18]. The NMR data were recorded from a fusion protein that contains a SKIP polypeptide connected to the PPIL1 protein by a flexible linker. Strikingly, this ensemble shows high similarity with the present docked model. For instance, the directionality of the SKIP peptide, the geometry of the binding pocket of PPIL1 and the function of Glu66 of SKIP as an anchoring point are very similar. However, [18] discerned an additional important site of interaction in the methyl group of Met76 of SKIP that was not found in the present study. The minimal PPIL1-binding peptide extracted from our array analysis does not include Met76, although a strong signal is observed on peptide A12 (Figure 1A), which includes Met76 at position 17. It is possible that a peptide array harboring peptides longer than 20 residues could have detected this additional anchoring point. Moreover, [18] describes an NOE distance constraint between Asp60 (numbered Asp184 in [18] due to the fusion construct) and Ile136 of PPIL1. This interaction was not found in our docked model, because Ile136 has not been described previously as a SKIP-interacting residue and was therefore not defined as an “active residue” in our docking run. In conclusion, our docking procedure succeeded in describing a large part of the PPIL1·SKIP interaction identifying a novel protein-protein interaction surface on a cyclophilin.

## Materials and Methods

### Cloning, protein expression and purification

A DNA fragment encoding full-length human PPIL1 protein was PCR-amplified from a human cDNA library (Marathon-Ready; Clontech, Mountain View, CA, USA) and cloned *via* BamHI/NotI restriction sites into the expression vector pGEX-6P1 (GE Healthcare, Freiburg, Germany). A synthetic DNA fragment encoding SKIP^43–79^, att sites, a TEV cleavage site and a stop codon were generated using the AssemblyPCRoligomaker [19] and a PCR protocol as suggested by the authors. The PCR product was inserted into the pET-G30 vector (EMBL, Heidelberg, Germany) using the Gateway “one tube” protocol following the manufacturer's instructions (Invitrogen, Carlsbad, US). For protein expression, plasmids were transformed into Rosetta 2 cells (Novagen, Madison, WI, US). Expression was performed at 16°C using an auto-inducing medium [20].

Bacterial cells were lyzed by using a microfluidizer (Microfluidics, Newton, MA, US). Clarified lysate was passed over glutathione-sepharose (GE Healthcare) according to the manufacturer's instructions. After cleavage of the affinity tag with PreScission protease, the sample was dialyzed against buffer A (20 mM Tris-HCl, pH 8.0, 120 mM NaCl, 1 mM DTT) and passed again over the affinity resin to remove remaining uncleaved species. Protein was collected in the flow-through and subjected to a final size exclusion chromatography (Superdex 75) in buffer A. Pooled fractions were concentrated to 20 mg/ml by ultrafiltration, aliquoted and flash-frozen in liquid nitrogen for storage at −70°C. For co-crystallization, complex formation with CsA (Sigma-Aldrich, Schnelldorf, Germany) was carried out in the absence of organic solvents by diluting the protein into a large volume of buffer A and addition of solid CsA in 5-fold molar excess as described [21]. The suspension was rotated gently overnight at 4°C, followed by removal of excess insoluble CsA by centrifugation. Supernatant protein·CsA complex was concentrated to 20 mg/ml (PPIL1) and used immediately for crystallization trials.

### Peptide array binding experiments

A total of 73 overlapping 20-mer peptides covering amino acids 49 to 140 of SKIP with a one amino acid offset were synthesized and assembled in duplicate as an array of spots on cellulose membranes (kind gift of Dr. C. Freund, FMP, Berlin, Germany). Membranes were washed for 10 min in 100% ethanol followed by three times washing for 10 min with 1× TBS buffer (130 mM NaCl, 2.8 mM KCl, 50 mM Tris-HCl, pH 8.0) to remove residual contaminants. Membranes were pre-blocked for 3 h in blocking buffer (5% (w/v) milk powder, 5% (w/v) saccharose in 1× TBS) and washed again for 10 min in 1× TBS. GST-PPIL1 fusion protein or GST alone were diluted in blocking buffer to a final concentration of 40 µg/ml. Membranes were incubated overnight at 4°C on a rocking platform. Unbound protein was removed by washing the membranes three times with 1× TBS. Subsequently, the membranes were incubated for 3 h at RT with anti-GST-antibody (GST (Z-5) sc-459, Santa Cruz Biotechnology, USA) diluted 1∶1000 in blocking buffer, washed three times with 1× TBS and incubated for 1.5 h with secondary antibody (peroxidase-conjugated α-rabbit, Jackson Immunoresearch, USA, diluted 1∶25000). The membranes were washed five times with 1× TBS and subsequently treated with ECL reagent (Perkin Elmer, MA, USA). Chemiluminescence was detected with a luminescent image analyzer (LAS-1000 Fujifilm) equipped with an appropriate set of filters.

### GST pull-down assay

GST and the GST-fusion polypeptide were captured on glutathione-coated sepharose beads (GE Healthcare) and washed extensively with TET-150 buffer (20 mM Tris-HCl, pH 7.5, 150 mM NaCl, 3 mM EDTA, 1 mM β-mercaptoethanol, 0.1% (v/v) Triton X-100). For each reaction, 50 µg of purified PPIL1 were incubated with 20 µl of protein-coated glutathione-beads for 2 h at 4°C on an overhead shaker. After extensive washing with either TET-150 or TET-1000 buffer (as TET-150, but 1 M NaCl), proteins were eluted by addition of SDS sample buffer and analyzed by SDS-PAGE.

### Crystallization and data collection

PPIL1 or PPIL1·CsA were mixed with synthetic SKIP peptides encompassing SKIP residues 55–74 and 44–74 (synthesized by Peptide Specialty Laboratories, Heidelberg, Germany) in twofold molar excess yielding a total final protein concentration of 12 mg/ml. Initial hits were found in trials of sample containing PPIL1, CsA and SKIP^44–74^. Despite extensive efforts, no conditions could be found that promoted the growth of single crystals. Thus, crystals from a plate cluster grown at 4°C in 0.1 M HEPES, pH 7.2, 0.7 M sodium acetate, 15 mM CdCl_2_ and 50 mM guanidine-HCl were singularized using an acupuncture needle. For data collection at cryogenic temperatures, the crystal was transferred into paratone N and flash-cooled in liquid nitrogen. Two datasets from the same crystal were collected at BESSY beamline 14.2 at different wavelengths. At a wavelength of 1.5 Å, data were collected to a resolution of 1.8 Å recording anomalous signal for Cd^2+^. After collecting a full ϕ-scan, the wavelength was tuned to the maximum photon flux (0.9 Å), which yielded a dataset to a maximum resolution of 1.15 Å. Diffraction data were processed with XDS [22].

### Structure solution and refinement

For SAD phasing of PPIL1·CsA, Shelx [23] was employed. Using diffraction data up to 1.8 Å resolution collected at a wavelength of 1.5 Å (for which I/σ(I)>2), ShelxD clearly located two Cd^2+^ ions per asymmetric unit with correlation coefficients of 39.0%/24.9% (CC_all_/CC_weak_). For density modification with ShelxE, the high-resolution data set was merged with the data set used for phasing. The pseudo-free correlation coefficient for the correct hand was clearly better than for the other hand (82% vs. 55%), indicating that a correct solution had been found. Automated model building with ARP/wARP resulted in a model comprising more than 90% of the expected protein residues. The FOM-weighted mean phase error relative to the final refined model was 23.4°. Refinement was conducted using the PHENIX package [24]. 5% of the data were set aside as a test set for calculation of the free R-factor. Ordered solvent was included as implemented in PHENIX. Coordinates of the refined structures and the structure factor amplitudes have been deposited in the Protein Data Bank (http://www.pdb.org, PDB ID 2x7k).

### Molecular docking

The coordinates for the PPIL1 protein were taken from the current crystal structure without modifications. Coordinates for a ten-residue SKIP peptide (sequence DGGAFPEIHV comprising SKIP residues 60 to 69) were generated by Pepfold [25] and geometry-minimized using the PHENIX package [24]. Restraints for the docking run were generated based on experimental data of the PPIL1-binding epitope of SKIP as mapped by peptide array analysis and SKIP-interacting residues of PPIL1 as mapped by HSQC-NMR by [1]. The charged SKIP residues Glu66 and His68 as well as the polar PPIL1 residues Tyr28, Asp89 and Arg131 were defined as “active residues”. Such “active residues” are residues required to have an interface contact of ambiguous distance. Passive residues were defined automatically as residues around active residues. Docking was carried out with default settings as semi-flexible simulated annealing followed by a final refinement in explicit water.

## Supporting Information

Figure S1 Multiple sequence alignment of human spliceosomal cyclophilins. The non-conserved Asp89 residue of PPIL1 is indicated by a grey arrow. The alignment was generated with T-Coffee (Notredame C, Higgins DG, Heringa J (2000) T-Coffee: A novel method for fast and accurate multiple sequence alignment. J Mol Biol 302: 205–217). Sequence numbering concerns the PPIase domains only, therefore it is distinct from the total residue numbers in the cases of PPIL2, PPWD1 and CypE (these proteins harbor domains preceding the PPIase domain).
(0.84 MB TIF)

Table S1 Selected bond lengths and angles of the Cd^2+^ coordination groups.
(0.04 MB DOC)
