# Supplementary figures and images for: The Crystal Structure of PPIL1 Bound to Cyclosporine A Suggests a Binding Mode for a Linear Epitope of the SKIP Protein
Source: PLoS One. 2010 Apr 2;5(4):e10013. doi: 10.1371/journal.pone.0010013 (PMC2848857; doi:10.1371/journal.pone.0010013)

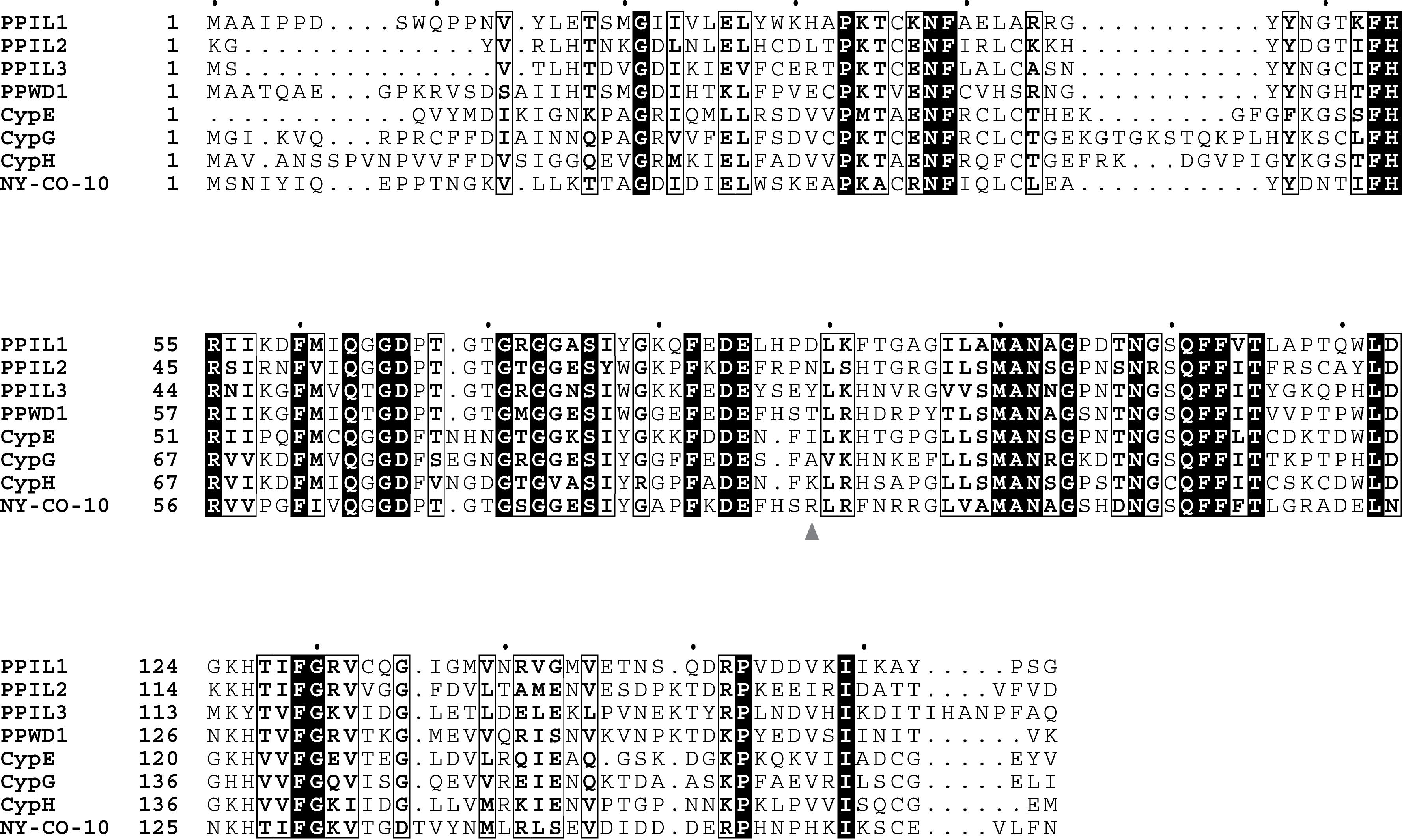

Supplement: Figure S1 — Multiple sequence alignment of human spliceosomal cyclophilins. The non-conserved Asp89 residue of PPIL1 is indicated by a grey arrow. The alignment was generated with T-Coffee (Notredame C, Higgins DG, Heringa J (2000) T-Coffee: A novel method for fast and accurate multiple sequence alignment. J Mol Biol 302: 205–217). Sequence numbering concerns the PPIase domains only, therefore it is distinct from the total residue numbers in the cases of PPIL2, PPWD1 and CypE (these proteins harbor domains preceding the PPIase domain). (0.84 MB TIF) [file pone.0010013.s001.tif]
