# Supplementary material for: The Crystal Structure of PPIL1 Bound to Cyclosporine A Suggests a Binding Mode for a Linear Epitope of the SKIP Protein
Source: PLoS One. 2010 Apr 2;5(4):e10013. doi: 10.1371/journal.pone.0010013 (PMC2848857; doi:10.1371/journal.pone.0010013)
Supplement: Table S1 — Selected bond lengths and angles of the Cd2+ coordination groups. (0.04 MB DOC) [file pone.0010013.s002.doc]

| *Bond lengths (Å)* |  | *Bond angles (°) for coordination number* | *5* | *4* |
| --- | --- | --- | --- | --- |
| Cd1 - His31 Nε2 | 2.29 | His31 Nε1 - Cd1 - Cys133 Sγ^a^ | 98.2 | 98.2 |
| Cd1 - Cys133 Sγ^a^ | 2.54 | His31 Nε1 - Cd1 - HOH58 | 99.3 | 99.3 |
| Cd1 - Asp89 Oδ2 | 2.32 | Cys133 Sγ^a^- Cd1 - HOH58 | 103.2 | 103.2 |
| Cd1 - Asp89 Oδ1 | 2.40 | His31 Nε1 - Cd1 - Asp89 Cγ^c^ |  | 123.4 |
| Cd1 - HOH58 | 2.44 | Cys133 Sγ^a^- Cd1 - Asp89 Cγ^c^ |  | 113.3 |
|  |  | HOH58 - Cd1 - Asp89 Cγ^c^ |  | 116.4 |
|  |  | His31 Nε1 - Cd1 - Asp89 Oδ1 | 97.6 |  |
|  |  | Cys133 Sγ^a^- Cd1 - Asp89 Oδ1 | 114.1 |  |
|  |  | HOH58 - Cd1 - Asp89 Oδ2 | 95.9 |  |
| Cd2 - His87 Nδ1^b^ | 2.20 | Glu26 Oε1 - Cd2 - Cys133 Sγ |  | 104.9 |
| Cd2 - Cys133 Sγ | 2.51 | Glu26 Oε1 - Cd2 - HOH2 |  | 94.9 |
| Cd2 - HOH2 | 2.49 | Glu26 Oε1 - Cd2 - His87 Nδ1 |  | 136.3 |
| Cd2 - Glu26 Oε1 | 2.15 | His87 Nδ1^b^- Cd2 - Cys133 Sγ |  | 112.2 |
|  |  | His87 Nδ1^b^- Cd2 - HOH2 |  | 93.4 |
|  |  | HOH2 - Cd2 - Cys133 Sγ |  | 109.9 |
| Cd1 - Cd2^a^ | 4.17 | Cd1 - Cys133 Sγ^a^- Cd2^a^ |  | 111.2 |

^a^ symmetry operator: 1/2 - x, y – 1/2 , -z

^b^ symmetry operator: 1/2 - x, y + 1/2, -z

^c^ considering the bidentate carboxyl as single ligand centered on Cγ
